# *Chimonanthus nitens* Oliv. Leaf Granule Ameliorates DSS-Induced Acute Colitis Through Treg Cell Improvement, Oxidative Stress Reduction, and Gut Microflora Modulation

**DOI:** 10.3389/fcimb.2022.907813

**Published:** 2022-06-27

**Authors:** Jia-Qi Huang, Si-Yi Wei, Nian Cheng, You-Bao Zhong, Fei-Hao Yu, Ming-Da Li, Duan-Yong Liu, Shan-Shan Li, Hai-Mei Zhao

**Affiliations:** ^1^Department of Postgraduate, Jiangxi University of Traditional Chinese Medicine (TCM), Nanchang, China; ^2^Laboratory Animal Research Center for Science and Technology, Jiangxi University of Traditional Chinese Medicine (TCM), Nanchang, China; ^3^Key Laboratory of Animal Model of Traditional Chinese Medicine (TCM) Syndromes of Depression, Jiangxi University of Traditional Chinese Medicine (TCM), Nanchang, China; ^4^College of Traditional Chinese Medicine, Nanchang Medical College (TCM), Nanchang, China; ^5^Formula-Pattern Research Center of Jiangxi University of Traditional Chinese Medicine (TCM), Nanchang, China; ^6^College of Traditional Chinese Medicine, Jiangxi University of Traditional Chinese Medicine (TCM), Nanchang, China

**Keywords:** *Chimonanthus nitens* Oliv. leaf granule, ulcerative colitis, oxidative stress, memory Treg, gut microflora

## Abstract

The rising incidence of ulcerative colitis has become a new challenge for public health. *Chimonanthus nitens* Oliv. leaf granule (COG) is a natural medicine used for the treatment of respiratory diseases, which has excellent anti-inflammatory and antioxidant effects. However, the therapeutic effect of COG in ulcerative colitis (UC) has not been reported. Here, the experimental colitis was treated with dextran sodium sulfate (DSS) and COG. After treatment with high (30 g/kg), medium (15 g/kg), and low (7.5 g/kg) doses of COG for 11 consecutive days, the body weight, disease activity index (DAI) score, colon length, colon weight index, and the pathological score of mice were effectively improved. COG significantly reduced the levels of inflammatory cytokines in UC mice *in vitro* and *in vivo* and restored the secretion levels of IL-6 and IL-10 in the colon. Meanwhile, compared to mice with colitis, COG-treated mice showed lower levels of MDA, MPO, NO, and eNOS and higher levels of GSH-Px and MAO, which indicated that oxidative stress damage in colitic mice was alleviated by COG. Moreover, less Th17 and more Tregs were observed in the COG-treated groups. In addition, COG improved the diversity and relative abundance of gut microflora in the colon of colitic mice, and *Lachnospiraceae_NK4A136_group* and *Lachnospiraceae_UCG-006* were obviously regulated at the genus level. In summary, COG has a protective effect on DSS-induced experimental colitis, mainly through inhibition of immune-inflammatory responses and oxidative stress and regulation of mTreg cell responses and intestinal flora composition.

## Introduction

Ulcerative colitis (UC) is a non-specific chronic relapsing inflammatory disorder that continuously damages the colonic mucosa from the rectal side, often leading to erosions and ulcers (Nakase et al., 2021). It mainly affects people between 18 and 50 years of age and seriously reduces the quality of life of patients (Li et al., 2017). Because of the unknown etiology and pathogenesis of UC, as well as its high recurrence rate and low cure rate, it is considered one of the top 10 intractable diseases in the world (Kaplan and Windsor, 2021). The pathogenesis of UC involves several mechanisms. Now, it is commonly accepted that immune abnormality, intestinal microecological imbalance, and oxidative stress are the three main suspected pathogenesis of UC (Xavier and Podolsky, 2007).

In the pathogenesis of UC, excessive inflammation and oxidative stress run through the pathogenesis of UC. Overproduction of IL-6 was reported to cause cytokine storm (Oldstone and Rosen, 2014; Oladele et al., 2020), which produced a large number of inflammatory cytokines and thus induced the increase of myeloperoxidase (MPO) level and the production of reactive oxygen species (Wu et al., 2005). Colonic cells were impaired by oxidative stress injury and further aggravated the inflammatory cell infiltration and injury (Adjouzem et al., 2020). Meanwhile, regulatory T-cell (Treg) imbalance could further aggravate the intestinal immune disorder of colitis. Some clinical studies have shown that a deficiency of Treg cells aggravates the clinical and endoscopic severity of UC (Takahashi et al., 2006; Kamikozuru et al., 2009), and it has been verified in animal experiments that the adoptive transfer of Treg cells effectively alleviated experimental colitis (Grayson et al., 2018). Moreover, the functional diversity and stability of intestinal flora in patients with UC are impaired, which suggests that intestinal flora is the basic factor involved in the inflammatory process of inflammatory bowel disease (IBD) (Hansen et al., 2010). Alleviating the clinical symptoms of UC patients by adding probiotics in the intestinal flora, as an emerging strategy to relieve chronic gut inflammation, has shown some promise in the treatment of UC (Vich Vila et al., 2018; Iheozor-Ejiofor et al., 2020). Interestingly, many natural medicines (such as daphnetin and dietary resveratrol) were reported to reduce pathogenic bacteria, increase probiotics, correct the imbalance of intestinal microbiota, and even change the metabolic characteristics of intestinal flora (Sartor, 2006; Ji et al., 2019; Li et al., 2020). Interestingly, such changes are causatively related to the enhanced development of Treg cells and the reduced proinflammatory Th17 cell differentiation. Therefore, reducing inflammatory damage, inhibiting oxidative stress, and regulating Treg and gut microflora structure can be regarded as potential therapeutic strategies for UC.

*Chimonanthus nitens* Oliv. leaf is a commonly used traditional Chinese medicine and has been known as one of the four famous medicinal herbs in Jiangxi Province (Chen et al., 2017b). Clinically, *C. nitens* Oliv. leaf is mostly used for upper respiratory disease, hand-foot-and-mouth disease, herpangina, and other diseases, with definite curative effect and safety (Wu et al., 2021). However, apart from the abovementioned diseases, traditionally, people often use *C. nitens* Oliv. leaf as a tea drink to alleviate diarrhea during the spring–summer. Many studies have shown that *C. nitens* Oliv. leaf has a variety of biologically active ingredients (including volatile oil, alkaloids, flavonoids, quercetin, rutin, and coumarin), with diverse pharmacological effects and antioxidant, immunomodulatory, hypolipidemic, and antitumor properties (Shu et al., 2019). These main active ingredients play biological activities to treat UC, which were reported by many researchers in a previous study (Vezza et al., 2016). For example, quercetin and rutin can reduce the production of proinflammatory mediators and inhibit NF-κB activity (Pavlick et al., 2002; Korhonen et al., 2005) and oxidative stress damage in UC (Zhang et al., 2017). Similarly, alkaloids have well-documented anti-inflammatory and antioxidant activities (Peng et al., 2019), which show contribution to the interaction with gut microflora, such as berberine (Liu et al., 2018).

*Chimonanthus nitens* Oliv. leaf granule (COG) has already been used in clinics. However, whether COG can be used to alleviate UC has not been reported. In this present study, we investigated the protective effects of *C. nitens* Oliv. leaf granule on mice with dextran sulfate sodium (DSS)-induced colitis and its potential mechanisms of action.

## Materials and Methods

### Drug Preparation

COG was purchased from Jiangxi, YouMei Pharmaceutical Co., Ltd. (Z20027113)(Nanchang, Jiangxi). Before use, it was dissolved in sterile water and heated at 70°C in a water bath for 20 min to fully release the active ingredients of the drug. The mother liquor of COG for the cell experiments was prepared as follows: The COG was weighed precisely and solvated in sterile PBS. Then, the mixture was treated for 20 min by using an ultrasonic cleaning machine with 300 W of power and heated in a water bath at 70°C for 30 min. After centrifugation at 2,000 rpm for 5 min, the supernatant was collected and filtered using a 0.22-μm syringe-filter unit. DSS (molecular weight: 36–50 kDa) was obtained from MP Biomedicals (Santa Ana, CA, USA). Mesalazine (batch number: 130407) was purchased from Sunflower Pharma (Jiamusi, China).

### Quantitative Liquid Chromatography Tandem-Mass Spectrometry Analysis of *Chimonanthus nitens* Oliv. Leaf Granule

Liquid chromatography tandem-mass spectrometry (LC-MS/MS) analysis was performed for the quality control of COG utilizing Thermo Fisher Scientific U3000 UHPLC (Bremen, Germany) coupled with an AB Sciex™ TripleTOF 5600+ (Framingham, MA, USA) at a column temperature of 35°C. The mobile phase (A: aqueous solution containing 0.1% of formic acid; B: acetonitrile solution containing 0.1% of formic acid) was delivered at a speed of 0.3 ml/min, and liquid chromatography separation was performed for 15 min per sample with linear gradient steps programmed as follows: 0–8 min (95%–5% B), 8–10 min (50%–50% B), 10–12 min (5%–95% B), and 12–15 min (95%–5% B).

### Mice

Male-specific pathogen-free (SPF) BALB/c mice (7–8 weeks of age, weighing 20–22 g) were purchased from GemPharmatech Co., Ltd. (Nanjing, China) (animal certificate number: SCXK 2018-0008) and housed in specific pathogen-free conditions (temperature: 21°C ± 2°C, humidity: 45% ± 10%, light: 12-h light–dark cycle). The protocol (permit number: JZLLSC20210065) was approved by the Animal Care and Use Committee of Jiangxi University of Traditional Chinese Medicine and conducted following the guidelines prescribed by the committee.

### DSS-Induced Colitis and Therapeutic Evaluation

**Figure 1:**
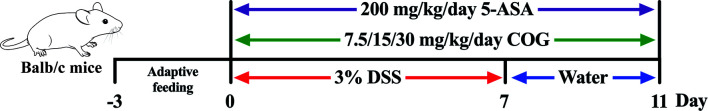
Colitis induction and *Chimonanthus nitens* Oliv. leaf granule (COG) administration.

All mice were acclimatized for 3 days prior to starting the study and then randomly divided into six groups with 10 mice in each group: normal group (Nor), DSS group (DSS), DSS+high-dose COG group (DSS+COG-H, 30 g/kg), DSS+medium-dose COG group (DSS+COG-M, 15 g/kg), DSS+low-dose COG group (DSS+COG-L, 7.5 g/kg), and DSS+mesalazine group (DSS+5-ASA, 200 mg/kg). To induce acute experimental colitis (**Figure 1**), BALB/c mice were administered with 3.0% (w/v) of DSS in their drinking water *ad libitum* for 7 days followed by 4 days of normal water (Wirtz et al., 2017). The mice of the normal group were provided with an equal amount of sterile water. From day 8 until the end of the experiment, mice in the treatment group were orally given COG and 5-ASA with corresponding concentrations, respectively. During the entire experimental period, all mice were weighed, their body weights were recorded, and the disease activity index (DAI) score, including fecal consistency score, blood stool score, and weight loss rate score, was calculated daily. DAI score was estimated using the following parameters: rate of weight loss (no significant loss, 0; loss of 1%–5%, 1; loss of 6%–10%, 2; loss of 11%–20%, 3; decline of more than 20%, 4), degree of loose stools [normal stools, 0; loose stools (dry), 2; loose stools or diarrhea (watery stools), 4], and degree of bleeding (no bleeding, 0; positive fecal occult blood, 2; blood in stools, 3; anal bleeding, 4).

### Pathological Histology Analysis

The colon samples were emptied of fecal contents and washed with cold phosphate-buffered saline (PBS, pH = 7.3). The distal colon samples were fixed in 4% paraformaldehyde (PFA) for 3 days at 4°C, dehydrated with grade ethanol solution (from 50% to 100%), and embedded in paraffin. The samples were cut into 4-μm-thick sections and then deparaffinized and rehydrated. The sections were stained with hematoxylin and eosin (H&E); subsequently, they were imaged with a biomicroscope. The microscopic scores were evaluated by two different pathologists blindly. Histopathological injury scores included inflammatory infiltrate and tissue damage. The points for infiltration were given as follows: 0, no infiltration; 1, increased number of inflammatory cells in the lamina propria; 2, inflammatory cells extending into the submucosa; and 3, transmural inflammatory infiltrates. For tissue damage, the points were as follows: 0, no mucosal damage; 1, discrete epithelial lesions; 2, erosions or focal ulcerations; and 3, severe mucosal damage with extensive ulceration extending into the bowel wall.

### Enzyme-Linked Immunosorbent Assay

The colon tissue of the mice was weighed and lysed using radioimmunoprecipitation assay (RIPA) buffer at a ratio of 1:10, homogenized with an electric homogenizer in ice water, incubated at 4°C for 30 min, and centrifuged at 12,000 rpm for 10 min to obtain the supernatant of the colon tissue homogenate. The total protein in each mouse was quantified with a total protein detection kit (Aidlab Biotechnologies Co., Ltd., Beijing, China). Cytokines including IL-4, IL-6, IL-10, and IL-1β were measured by commercial enzyme-linked immunosorbent assay (ELISA) kits following the manufacturer’s protocol, and the absorbance values of each sample were detected using a microplate reader (Thermo, Varioskan, MA, USA). Then, the concentrations of the cytokines were obtained according to standard curves.

### Antioxidant Activity Determination of the Colon Tissue

The colon tissue was weighed and homogenized with sterile saline (1:9) on ice using a handheld tissue homogenizer for 45 s at 12,000 rpm. Then, the homogenate was centrifuged at 2,500 rpm for 10 min at 4 C. The supernatant was collected and diluted with sterile saline (1:15), and the levels of malondialdehyde (MDA), glutathione (GSH), nitric oxide (NO), endothelial nitric oxide synthase (eNOS), monoamine oxidase (MAO), and myeloperoxidase (MPO) in a 50-μl sample were examined according to the manufacturer’s instructions. The determination kits were provided by Nanjing Jiancheng Bioengineering Institute (Nanjing, China).

### Flow Cytometric Analysis

In a sterile environment, the abdominal cavity was rapidly opened and the spleen was isolated on ice, then placed in a Petri dish, and minced with ophthalmic scissors. Subsequently, 1 ml of 1640 cell culture solution was added, and the spleen was gently ground with a 5-ml syringe core, followed by adding another 1 ml of 1640. The cell mixture was filtered with a 70-μm cell sieve and centrifuged at 300×*g* for 5 min, the supernatant was removed, and the red cells were eliminated by lysis in 1 ml of erythrocyte lysis buffer. Then, the cells were rinsed twice and centrifuged at 350×*g* for 5 min. The supernatant was discarded, and the cells were resuspended in 100 μl of stain buffer. The cell suspension was stimulated with a leukocyte activation cocktail (InvivoGen, 00-4975-93) at 37 C in 5% CO_2_ for 2–4 h. Then, 1 μg of FcR blocking sealant was added and incubated at 4°C for 8 min. Furthermore, the cells were stained with cell-surface antibodies and incubated for 15 min at room temperature in the dark. The cells were washed with 1 ml of stain buffer twice, the supernatant was discarded, and 500 μl of stain buffer was added to resuspend the cells. Then, the cells were fixed and permeabilized using a Cytofix/Cytoperm kit (BD Biosciences, Franklin Lakes, NJ, USA), followed by incubation with intracellular and intranuclear antibodies for 40 min at 4 C in the dark. Finally, these stained cells were detected using a FACSCanto II flow cytometer (BD Biosciences, NJ, USA). The following mAbs were used: BV510 rat anti-mouse CD4 (1:200), PerCP rat anti-mouse CD45 (1:100), Alexa Fluor 647 rat anti-mouse CCR7 (1:100), BV421 rat anti-mouse Foxp3 (1:100), PE rat anti-mouse IL-17A (1:100), and Alexa Fluor 488 rat anti-mouse IL-10 (1:100) (BD Biosciences). The cells were washed, resuspended in 500 μl of stain buffer, and finally detected using a FACSCanto II flow cytometer (BD Biosciences, NJ, USA). The limits for the quadrant markers were set based on negative populations and isotype controls. The analysis of the acquired data was performed with the FlowJo software (FlowJo 10.4).

### Fecal Microbial Community Analysis

Colonic contents of all mice were collected in cryopreservation tubes and preserved at −80°C. Microbial diversity analysis was conducted according to the procedures of Majorbio Bio-Pharm Technology (Shanghai, China). The genomic DNA was extracted from fecal samples using the QIAamp DNA Stool Mini Kit (Qiagen, Valencia, CA, USA), according to the manufacturer’s instructions. The extracted DNA was used as a template to amplify the V3–V4 region of the bacterial 16S rRNA gene with the following primers: 338F (5′-ACTCCTACGGGAGGCAGCAG-3′) and 806R (5′-GGACTAC HVGGGTWTCTAAT-3′). The PCR reaction consisted of 4 μl of 5× FastPfu buffer, 2 μl of 2.5 mM dNTPs, 0.8 μl of forward primer (5 μM), 0.8 μl of reverse primer (5 μM), 0.4 μl of FastPfu polymerase (Trans-Start FastPfu DNA Polymerase, TransGen Biotech, Beijing, China), and 10 ng of template DNA. The amplification was performed on an Applied Biosystems PCR system (GeneAmp 9700, ABI, USA) as follows: 3 min of denaturation at 95°C, 27 cycles of 30 s at 95°C, 30 s at 55°C, and 45 s at 72°C, with a final elongation at 72 C for 10 min. The PCR products were purified using the AxyPrep DNA gel extraction kit (Axygen, Union City, USA). High-throughput sequencing was carried out on a MiSeq platform (Illumina, San Diego, CA, USA) according to the standard protocols by Majorbio Bio-Pharm Technology. The quality control of the raw sequence reads, deposited in the NCBI Sequence Read Archive (SRA) database (accession number: PRJNA655607), was conducted by FastQC on FastQ files. The operational taxonomic units (OTUs) were clustered with 97% similarity cutoff using UPARSE (version 7.1). The taxonomy of each 16S rRNA gene sequence was assigned by the Ribosomal Database Project (RDP) Classifier algorithm (http://rdp.cme.msu.edu/) against the SILVA (SSU123) 16S rRNA database using a confidence threshold of 70%. The Shannon index was calculated using Mothur (version v.1.30.1) to evaluate α-diversity. Partial least squares discriminant analysis (PLS-DA) was performed using Mothur, and statistical analysis was performed based on the values of COMP4. Linear discriminant analysis (LDA) coupled with effect size (LEfSe) measurements (based on non-parametric factorial Kruskal–Wallis sum-rank test and the Wilcoxon rank-sum test) was used to identify taxa that were significantly different (biomarkers) among groups, with *p <*0.05 and an LDA score threshold of 4. Microbial difference analysis and correlation analysis were performed using I-Sanger (Majorbio Bio-Pharm Technology Co., Ltd.; www.i-sanger.com).

### Cell Culture and Treatment

The murine macrophage RAW264.7 cells (Procell Life Science and Technology Co., Ltd., Wuhan, China) were cultured in DMEM medium containing 10% (v/v) of FBS and 1% (v/v) of penicillin–streptomycin (100 units/ml of penicillin, 100 μg/ml of streptomycin) at 37 C in a humidified incubator with 5% of CO_2_/95% of air atmosphere and subcultured as needed using trypsin–EDTA. The culture medium was changed every 2–3 days.

### Cytotoxicity Assay on RAW264.7 Cells

The cell viability was determined by the Cell Counting Kit-8 (CCK-8) assay (AbMole BioScience, USA). Briefly, RAW264.7 macrophages were seeded in 96-well plates (1 × 10^5^ cells/well). After adhering for 4 h, the cells were co-cultured with 2, 4, 6, and 8 mg/ml of COG for 24 h. After co-cultured with COG, 10 μl of the CCK-8 reagent was added to each well and the plates were incubated at 37 C in a humidified incubator with 5% of CO_2_/95% of air atmosphere for 2 h. A microplate reader (Thermo Fisher, USA) was used to measure the absorbance at a wavelength of 450 nm. According to the OD value, the cell viability and half-maximum inhibitory concentration (IC_50_) were evaluated by analysis using the software GraphPad Prism 8.0. Each experiment was performed in triplicate.

### Anti-Inflammatory Assay and Real-Time PCR on RAW264.7 Cells

The RAW264.7 macrophages were seeded in a six-well plate at a density of 1 × 10^6^ cells/well and allowed to reach confluence in this experiment. COG (0, 2, 4, 6, 8 mg/ml) and 1 μg/ml of LPS (*Escherichia coli* 055:B5, Sigma Aldrich, USA) were added to the cells in the different dose groups. After being treated with LPS and COG for 24 h, the cell culture medium from the cells was collected and centrifuged to remove any debris. Enzyme kits were applied for the determination of the proinflammatory cytokines TNF-α (BMS607-3, Invitrogen, USA), IL-6 (MM-0163M1, Meimian, China), and IL-1β (MM-0040M1, Meimian, China) in the RAW264.7 macrophages according to the manufacturers’ instructions. All experiments were carried out in five replicates.

In order to investigate the RNA expression levels of IL-1β, IL-6, and TNF-α, total RNA was isolated from the cells treated with COG (2, 4, 6, 8 mg/ml) or LPS (1 μg/ml) for 24 h and reverse-transcribed to cDNA using M5 Super plus qPCR RT kit (Mei5 Biotechnology, China) based on the manufacturer’s protocol. Then, the IL-1β, IL-6, TNF-α, and GAPDH cDNA were amplified by 2× Real-time PCR SuperMix (Mei5 Biotechnology, China). cDNA was normalized to the same concentration. The PCR reaction was conducted on a bioanalyzer (Roche LightCycler 96 instrument) programmed as follows: 95 C for 60 s and 45 cycles of 95 C for 15 s, 60 C for 15 s, and 72 C for 45 s. The primer sequences are listed in **Table 1**. *GAPDH* was used as the housekeeping gene and the expression level of the target gene was calculated using the 2^−ΔΔCt^ method.

**Table 1:** Primers used in this study.

Primers	5′–3′ (forward)	5′–3′ (reverse)
*IL-1β*	CTGAACTCAACTGTGAAATGC	TGATGTGCTGCTGCGAGA
*IL-6*	GTTCTCTGGGAAATCGTGGA	TGTACTCCAGGTAGCTA
*TNF-α*	GCCTCTTCTCATTCCTGCTT	TGGGAACTTCTCATCCCTTTG
*GAPDH*	TGGTGAAGGTCGGTGTGAAC	TGAATTTGCCGTGAGTGGAG

### Statistical Analysis

All the data were presented as mean ± standard error of the mean (SEM), and GraphPad Prism software version 8.3.1 (GraphPad Software Inc., La Jolla, CA, USA) was used for the statistical analysis and plotting. One-way analysis of variance (ANOVA), followed by the Tukey–Kramer multiple comparisons test, was used to analyze the significant difference among the six groups. All *p*-values <0.05 were considered statistically significant.

## Results

### Quantitative Analysis of COG by LC-MS/MS

**Figure 2:**
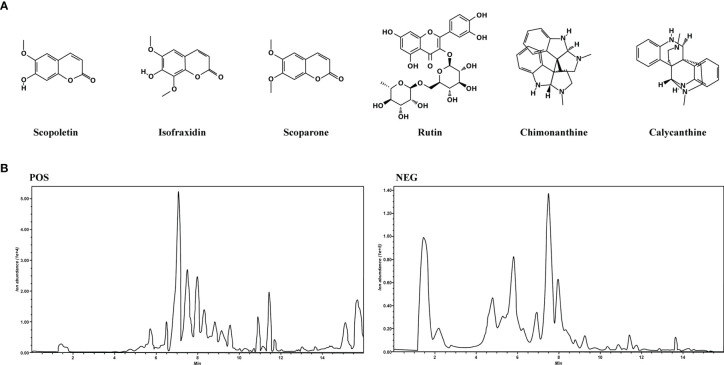
Analysis of the components of *Chimonanthus nitens* Oliv. leaf granule. **(A)** The chemical structure of scopoletin, isofraxidin, scoparone, rutin, chimonanthine, and calycanthine. **(B)** Total ion chromatogram of *C. nitens* Oliv. leaf granule in positive and negative ion modes.

LC-MS/MS analysis was performed to establish the quality control of COG by identifying and quantifying ingredients, namely, scopoletin, isofraxidin, scoparone, rutin, chimonanthine, and calycanthine. The structures of the aforementioned compounds are displayed in **Figure 2A**, and the general ion flow chromatogram of COG is shown in **Figure 2B**. The scopoletin, isofraxidin, scoparone, rutin, chimonanthine, and calycanthine contents in COG were 0.16, 0.11, 0.08, 0.50, 0.08, and 0.32 μg/ml, respectively (**Table 2**).

**Table 2:** Quantification of the components of COG.

Component name	Scopoletin	Isofraxidin	Scoparone	Rutin	Chimonanthine	Calycanthine
Content (μg/ml)	0.16	0.11	0.08	0.50	0.08	0.32

### COG Alleviated DSS-Induced Colitis

**Figure 3:**
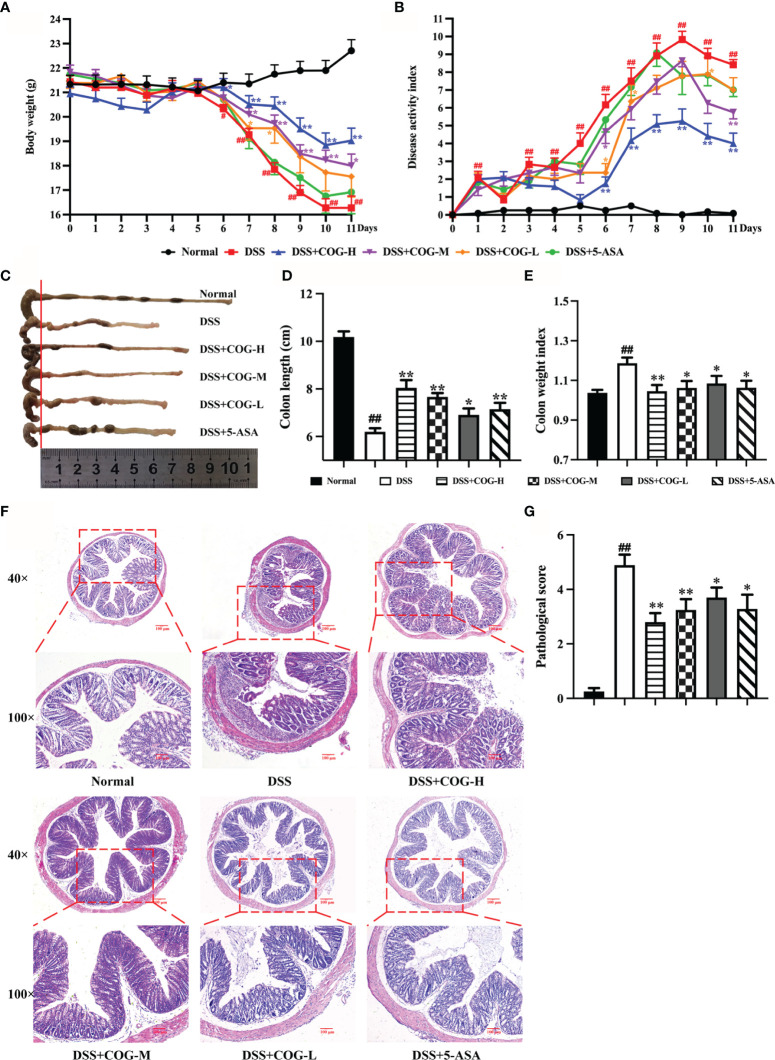
COG treatment ameliorated DSS-induced experimental colitis. **(A)** Body weight. **(B)** Disease activity index (DAI) score. **(C)** Representative pictures of colon gross appearance and colon length. **(D)** Colon length. **(E)** Colon weight index. **(F)** Representative microscopic pictures of H&E-stained sections (magnifications: ×40 and ×100). **(G)** Pathological injury score. Data were presented as mean ± SEM (*n* = 8–12). ^#^*p *< 0.05 and ^##^*p *< 0.01 compared to the normal group; ^*^p < 0.05 and **p < 0.01 compared to the DSS group.

Ulcerative colitis was successfully induced in all mice after DSS administration for 7 days, which was evidenced by the continuous body weight loss, DAI score increase, shortened colon length, and higher colon weight index (**Figures 3A–E**, respectively), compared with animals without DSS treatment. Compared with DSS treatment, the administration of high- and medium-dose COG significantly ameliorated DSS-induced colitis, as evidenced by the markedly reduced weight loss (**Figure 3A**), lower DAI score (**Figure 3B**), and significant relief of colonic shortening and colon weight index (**Figures 3C–E**). Although the low-dose COG did not present an obvious effect on weight loss prevention (**Figure 3A**) and DAI score increase (**Figure 3B**), it mitigated colon length shortening and colon weight index increase (**Figures 3C–E**). Additionally, the H&E-stained DSS sections revealed a destruction of colonic tissue with histopathological changes in the mucosal, submucosal, and muscular layers and colonic wall. The mucosa was ulcerated with an abundant inflammatory infiltrate in the submucosa; also, the colonic wall was thickened. Conversely, the different DSS+COG dose groups all exhibited less inflammatory cell infiltration, relatively intact colonic architecture, less mucosal damage, and lower histology score than the DSS group (**Figures 3F, G**). These results indicated that COG effectively alleviated experimental colitis induced by DSS.

### COG Relieved the Inflammation Response *In Vivo* and *In Vitro*

**Figure 4:**
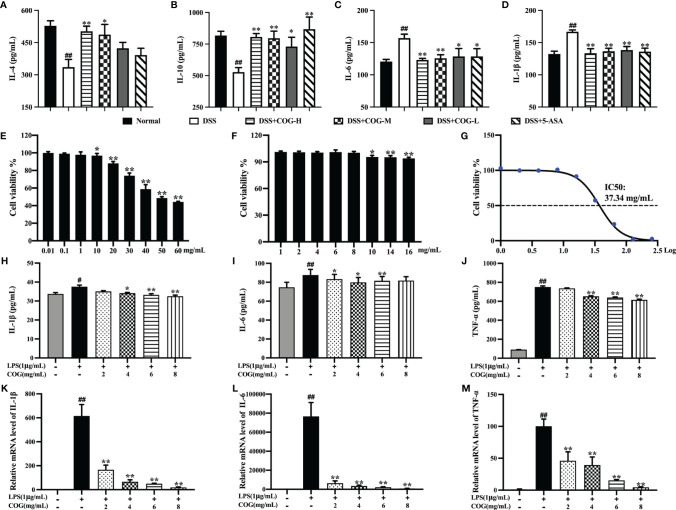
Anti-inflammatory effects of COG *in vivo* and *in vitro*. Cytokine levels of **(A)** IL-4, **(B)** IL-10, **(C)** IL-6, and **(D)** IL-1β in mice colon. **(E, F)** Cell viability of COG on RAW264.7 cells. **(G)** IC_50_ of COG on RAW264.7 cells. Inflammatory cytokine secretion levels of **(H)** IL-1β, **(I)** IL-6, and **(J)** TNF-α on RAW264.7 cells. The relative mRNA expression levels of **(K)** IL-1β, **(L)** IL-6, and **(M)** TNF-α on RAW264.7 cells. Data were presented as mean ± SEM (*n* = 6–12). ^#^*p *< 0.05 and ^##^*p *< 0.01 compared to the normal group; ^*^p < 0.05 and ^**^p < 0.01 compared to the DSS or LPS group.

To investigate whether COG intervention could ameliorate colitis by modulating the inflammatory response, inflammatory cytokines were analyzed in the colon tissue. IL-4 and IL-10 concentrations in the DSS group were significantly lower than those in the normal group (**Figures 4A, B**), while IL-6 and IL-1β were higher (**Figures 4C, D**), which suggested that DSS successfully induced inflammation in mice colon. Furthermore, compared to the DSS group, COG treatment significantly upregulated the concentrations of IL-4 and IL-10 in colon tissue (**Figures 4A, B**), and a decrease in the concentrations of IL-6 and IL-1β was observed in the COG group (**Figures 3A, C**).

The cytotoxicity of COG was assessed first. When the concentration of the COG solution exceeded 10 mg/ml, COG had a significant effect on the relative proliferation rate of cells and this relative proliferation rate of cells decreased with the continuous increase of COG concentration (**Figure 4E**). When the concentration was below 10 mg/ml, the relative proliferation rate of RAW264.7 cells was close to 100%, indicating that COG had no significant toxic effect on the cells at the dose below 10 mg/ml (**Figures 4E, F**). Our experiments showed that the IC_50_ value of COG on RAW264.7 cells was 37.34 mg/ml (**Figure 4G**). Therefore, the concentrations of 2, 4, 6, and 8 mg/ml were chosen as optimal for the subsequent treatments.

The anti-inflammatory activity of COG *in vitro* was performed in LPS-induced RAW264.7 macrophages. Compared to the control group, LPS stimulated remarkably the secretion of IL-1β, IL-6, and TNF-α (**Figures 4H–J**). After treatment with different concentrations of COG, the secretion levels of these three inflammatory cytokines all showed downward trends (**Figures 4H–J**). Among them, 2 mg/ml of COG could only significantly reduce the secretion level of IL-6 (**Figure 4I**). At the concentrations of 4 and 6 mg/ml, COG significantly decreased the secretion levels of IL-1β, IL-6, and TNF-α (**Figures 4H–J**). When the concentration of COG was 8 mg/ml, the levels of IL-1β and TNF-α in RAW264.7 cells were significantly reduced (**Figures 4H, J**). Similarly, LPS could significantly upregulate the IL-1β, IL-6, and TNF-α mRNA expression levels on RAW264.7 cells. After treatment with different concentrations of COG, the mRNA expression levels of IL-1β, IL-6, and TNF-α were decreased remarkably (**Figures 4K–M**). The results above indicated that COG effectively reduced the inflammation of DSS-induced colitis in mice *in vivo* and *in vitro*.

### COG Improved the DSS-Induced Oxidative Stress

**Figure 5:**
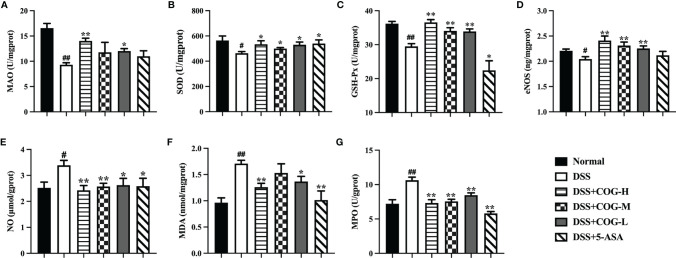
Antioxidant activity of COG in colitic mice. **(A)** Monoamine oxidase (MAO) activity changes. **(B)** Superoxide dismutase (SOD) activity. **(C)** Glutathione peroxidase (GSH-Px) activity changes. **(D)** Endothelial nitric oxide synthase (eNOS) production. **(E)** Nitric oxide (NO) production. **(F)** Malondialdehyde (MDA) production. **(G)** Myeloperoxidase (MPO) activity changes. Data were presented as mean ± SEM (*n* = 8–12). ^#^*p *< 0.05 and ^##^*p *< 0.01 compared to the normal group; ^*^p < 0.05 and ^**^p < 0.01 compared to the DSS group.

To further assess whether COG treatment has an antioxidant effect on DSS-induced mice, we examined several indicators involved in oxidative stress. Compared to the normal group, the DSS group showed a significant decrease in the levels of MAO, superoxide dismutase (SOD), glutathione peroxidase (GSH-Px), and eNOS (**Figures 5A–D**), while the levels of NO, MDA, and MPO were significantly increased in the DSS group (**Figures 5E–G**). After the administration of COG, a significant increase was observed in the levels of MAO, SOD, GSH-Px, and eNOS in the high- and low-dose COG groups (**Figures 5A–D**), while in the medium-dose COG group, an obvious effect was found in the levels of SOD, GSH-Px, and eNOS (**Figures 5B–D**). The concentrations of NO, MDA, and MPO were significantly reduced in the COG groups except in the medium-dose COG group, where no significant effect on the level of MDA was found (**Figures 5E–G**). The above findings confirmed that COG treatment alleviated oxidative stress damage in colon tissues, but this was not evident in a dose-dependent manner.

### COG Regulated the T Lymphocyte Subsets

**Figure 6:**
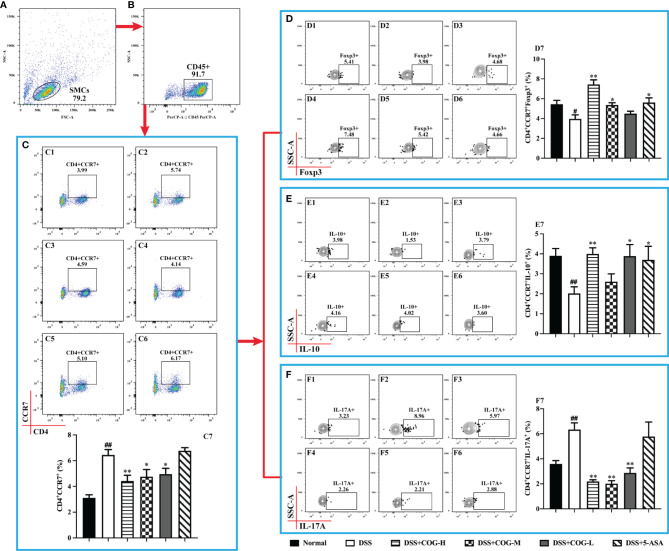
COG regulated CCR7^+^CD4^+^ T cells in mice with colitis. **(A)** Spleen mononuclear cells (SMCs). **(B)** CD45^+^ cells. **(C)** Flow cytometry analysis of CCR7^+^ memory T cells: C1–C6 represent CCR7^+^ memory T cells in the normal, DSS, DSS+COG-H, DSS+COG-M, DSS+COG-L, and DSS+5-ASA groups, respectively; C7: statistical analysis of CCR7^+^ memory T-cell frequencies in these six groups. **(D)** Flow cytometry analysis of Foxp3^+^CCR7^+^ memory T cells: D1–D6 represent Foxp3^+^CCR7^+^ memory T cells in the normal, DSS, DSS+COG-H, DSS+COG-M, DSS+COG-L, and DSS+5-ASA groups, respectively; D7: statistical analysis of Foxp3^+^CCR7^+^ memory T-cell frequencies in these six groups. **(E)** Flow cytometry analysis of IL-10^+^CCR7^+^ memory T cells: E1–E6 represent IL-10^+^CCR7^+^ memory T cells in the normal, DSS, DSS+COG-H, DSS+COG-M, DSS+COG-L, and DSS+5-ASA groups, respectively; E7: statistical analysis of Foxp3^+^CCR7^+^ memory T-cell frequencies in these six groups. **(F)** Flow cytometry analysis of IL-17A^+^CCR7^+^ memory T cells: F1–F6 represent IL-17A^+^CCR7^+^ memory T cells in the normal, DSS, DSS+COG-H, DSS+COG-M, DSS+COG-L, and DSS+5-ASA groups, respectively; F7: statistical analysis of IL-17A^+^CCR7^+^ memory T-cell frequencies in these six groups. Data were presented as mean ± SEM (*n* = 8–12). ^#^*p *< 0.05 and ^##^*p *< 0.01 compared to the normal group; ^*^p < 0.05 and ^**^p < 0.01 compared to the DSS group.

CCR7, a chemokine receptor that controls homing to secondary lymphoid organs, was highly expressed on mature DCs as well as naive and central memory T cells (Sallusto et al., 1999). In this study, we gated on CD45+ cells of Spleen mononuclear cells (SMCs) (**Figures 6A, B**) the frequency of CD4^+^CD45^+^CCR7^+^ cells was significantly higher in the DSS group than in the normal group (**Figure 6C**). The frequency of CD4^+^CD45^+^CCR7^+^ cells significantly decreased in the different COG treatment groups compared to the DSS group (**Figure 6C**). Based on CD4^+^CD45^+^CCR7^+^ cells, the expression of cytokine IL-17A (**Figure 6D**) was upregulated, while the levels of IL-10 (**Figure 6E**) and Foxp3 (**Figure 6F**) were downregulated, which indicated that DSS induced inflammation in the colon of mice. By contrast, COG intervention reduced the frequency of CD4^+^CD45^+^CCR7^+^ and CD4^+^CD45^+^CCR7^+^IL-17A^+^ cells and upregulated the frequency of CD4^+^CD45^+^CCR7^+^IL-10^+^ and CD4^+^CD45^+^CCR7^+^Foxp3^+^ cells.

**Figure 7:**
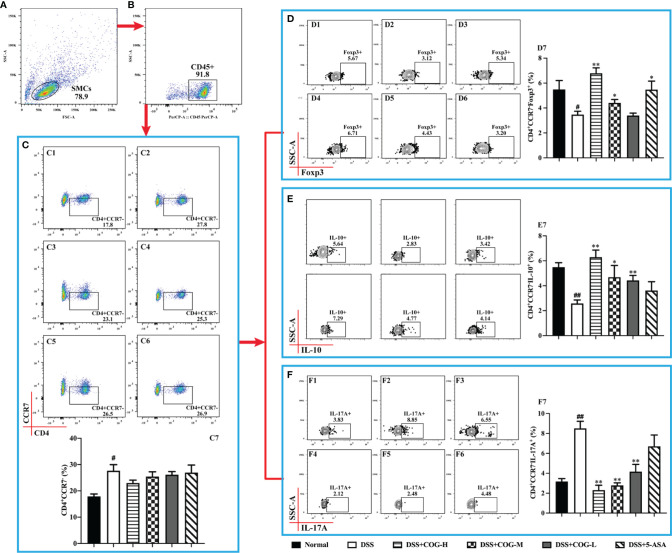
COG regulated CCR7^−^CD4^+^ T cells in mice with colitis. **(A)** Spleen mononuclear cells (SMCs). **(B)** CD45^+^ cells. **(C)** Flow cytometry analysis of CCR7^−^ memory T cells: C1–C6 represent CCR7^−^ memory T cells in the normal, DSS, DSS+COG-H, DSS+COG-M, DSS+COG-L, and DSS+5-ASA groups, respectively; C7: statistical analysis of CCR7^−^ memory T-cell frequencies in these six groups. **(D)** Flow cytometry analysis of Foxp3^+^CCR7^−^ memory T cells: D1–D6 represent Foxp3^+^CCR7^−^ memory T cells in the normal, DSS, DSS+COG-H, DSS+COG-M, DSS+COG-L, and DSS+5-ASA groups, respectively; D7: statistical analysis of Foxp3^+^CCR7^−^ memory T-cell frequencies in these six groups. **(E)** Flow cytometry analysis of IL-10^+^CCR7^−^ memory T cells: E1–E6 represent IL-10^+^CCR7^−^ memory T cells in the normal, DSS, DSS+COG-H, DSS+COG-M, DSS+COG-L, and DSS+5-ASA groups, respectively; E7: statistical analysis of Foxp3^+^CCR7^−^ memory T-cell frequencies in these six groups. **(F)** Flow cytometry analysis of IL-17A^+^CCR7^−^ memory T cells: F1–F6 represent IL-17A^+^CCR7^−^ memory T cells in the normal, DSS, DSS+COG-H, DSS+COG-M, DSS+COG-L, and DSS+5-ASA groups, respectively; F7: statistical analysis of IL-17A^+^CCR7^−^ memory T-cell frequencies in these six groups. Data were presented as mean ± SEM (*n* = 8–12). ^#^*p *< 0.05 and ^##^*p *< 0.01 compared to the normal group; ^*^p < 0.05 and **p < 0.01 compared to the DSS group.

Effector memory T cells that had a low expression of CCR7 mediated inflammatory reactions or cytotoxicity in peripheral tissues, thus rapidly containing invasive pathogens (Reinhardt et al., 2001). Moreover, CCR7 deficiency led to the accumulation of effector/memory-like Tregs at the inflammatory site (Menning et al., 2007). In this study, when gated on CD45+ cells of spleen mononuclear cells, the number of CD4^+^CD45^+^CCR7^−^ cells (**Figure 7C**) in the DSS group, which performed effector cell functions, was significantly higher than that in the normal group, indicating that inflammation accumulated in mice. Meanwhile, the expression of IL-17A on them (**Figure 7F**) was significantly increased. After treatment with COG and 5-ASA, compared with DSS treatment, CD4^+^CD45^+^CCR7^−^ cells (**Figure 7C**) decreased to some extent, but it was not statistically significant. However, the cytokines they secreted were apparently regulated. The level of IL-17A was remarkably upregulated, while the expression levels of IL-10 and Foxp3 were downregulated in the different doses of the COG treatment groups and the 5-ASA group (**Figures 7D, E**). The results showed that COG could effectively regulate the CCR7^−^CD4^+^ T cells in DSS-induced experimental colitis.

### COG Modulated the Composition of the Gut Microflora

**Figure 8:**
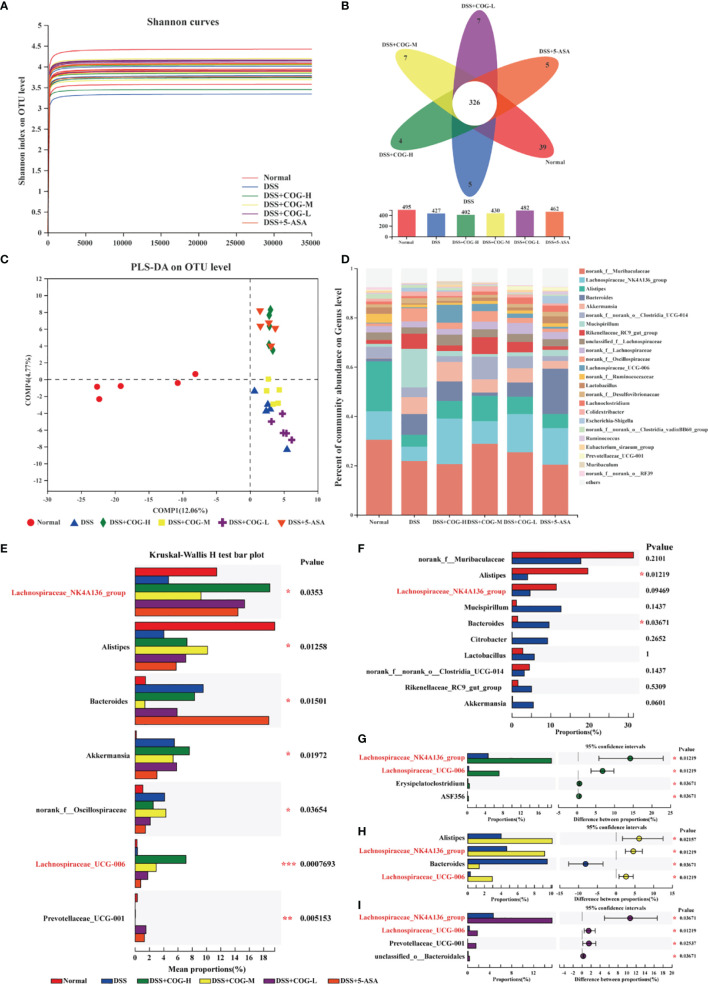
COG improved the composition of gut flora in colitic mice. **(A)** Shannon index of operational taxonomic unit (OTU) level. **(B)** The Venn diagram depicts OTUs that differed in each group. **(C)** PLS-DA of OTU level of taxonomy of gut flora in mice. **(D)** The percentage of community abundance at the genus level. **(E)** Top 7 bacterial rankings of microflora abundance at the genus level. **(F)** Comparisons between the normal group and the DSS group at the genus level. **(G)** Comparisons between the DSS group and the DSS+COG-H group at the genus level. **(H)** Comparisons between the DSS group and the DSS+COG-M group at the genus level. **(I)** Comparisons between the DSS group and the DSS+COG-L group at the genus level. *n* = 5.

The fecal microbial populations of mice in the normal, DSS, DSS+COG-H, DSS+COG-M, DSS+COG-L, and DSS+5-ASA groups were analyzed using 16S gene sequencing. The Shannon index curves of all samples tended to flatten (**Figure 8A**), indicating that the amount of sequencing data obtained from each sample was large enough, and the results demonstrated that the majority of the diversity was captured in all samples. The overlaps between the six groups were shown by the Venn diagram. There were 24, 15, 3, 3, 6, and 11 distinct OTUs in the normal, DSS, DSS+COG-H, DSS+COG-M, DSS+COG-L and DSS+5-ASA groups, respectively, whereas a total of 378 OTUs were shared among these groups (**Figure 8B**). Partial least squares discrimination analysis (PLS-DA) (**Figure 8C**) showed that these six groups had different compositions of gut microflora. The normal group showed an obvious distinction from the other groups; the DSS+COG-H and DSS+5-ASA groups shared the same quadrants; and the DSS, DSS+COG-M, and DSS+COG-L groups were clustered into different quadrants. PLS-DA also showed that the difference between the groups was greater than that within the groups (**Figure 8C**).

Compared to the percentage of community abundance at the genus level of the normal group, *Lachnospiraceae_NK4A136_group* and *Alistipes* of the DSS group were significantly downregulated, while *Mucispirillum* was significantly upregulated (**Figure 8D**). After COG treatment, the percentage of community abundance at the genus level of *Lachnospiraceae_NK4A136_group* and *Alistipes* was upregulated, while *Mucispirillum* was downregulated (**Figure 8D**). To determine the role of COG in regulating the gut flora of colitic mice, differential analysis among these four groups at the genus level (**Figure 8E**) was performed. A multispecies difference test at the genus level was performed on the data from the top 7 bacterial rankings of microflora abundance in the samples to detect species differences in microflora community abundance between groups, namely, *Lachnospiraceae_NK4A136_group*, *Alistipes*, *Bacteroides*, *Akkermansia*, *norank_f_Oscillospiraceae*, *Lachnospiraceae_UCG-006*, and *Prevotellaceae_UCG-001* (**Figure 8E**). Comparisons were made between every two groups, and it was found that there was a significant decrease of *Alistipes* in the DSS group compared to the normal group and that *Bacteroides* increased apparently in the DSS group. Also, the level of *Lachnospiraceae_NK4A136_group* in the DSS group decreased substantially, albeit without reaching statistical significance (**Figure 8F**). Interestingly, *Lachnospiraceae_NK4A136_group* and *Lachnospiraceae_UCG-006* decreased obviously in all the COG treatment groups compared to the DSS group (**Figure 8G–I**). The aforementioned results showed that COG improved the gut composition of microflora in colitic mice.

### The Correlation Among COG, Gut Microflora, and T Lymphocyte Subsets

**Figure 9:**
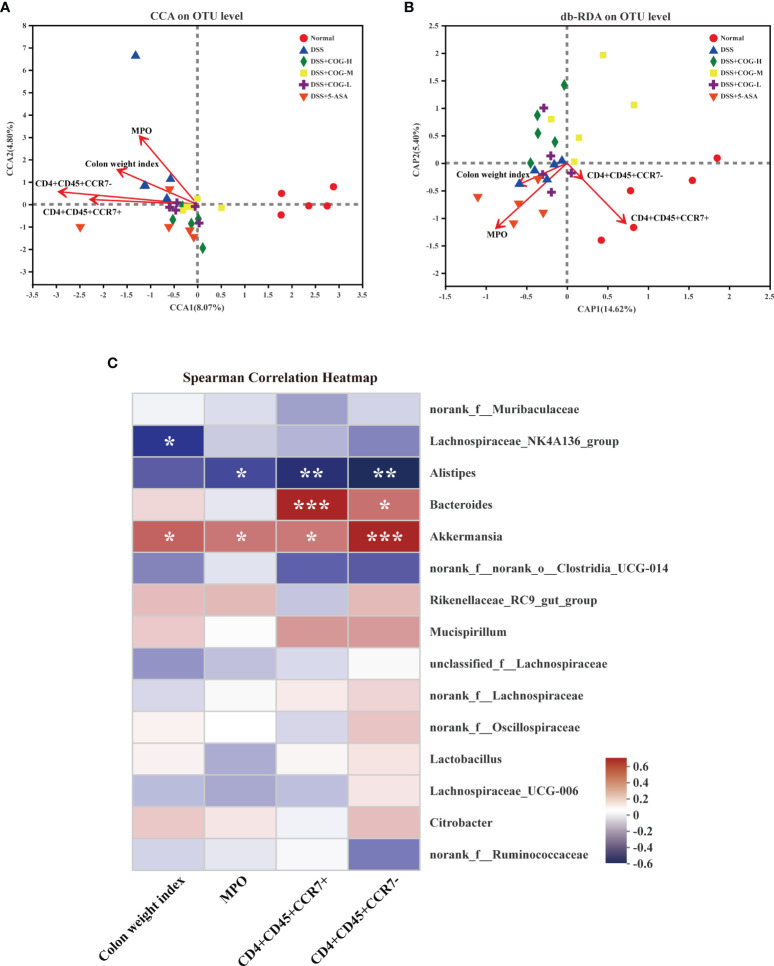
COG regulated the correlation among oxidative stress response, CCR7^+^/CCR7^−^ T lymphocytes, and gut microflora in colitic mice. **(A)** Correlation among colonic index, MPO, and CCR7^+^/CCR7^−^ T lymphocytes displayed by canonical correlation analysis (CCA). **(B)** Correlation among colonic index, MPO, and CCR7^+^/CCR7^−^ T lymphocytes displayed by distance-based redundancy analysis (db-RDA). **(C)** Spearman’s correlation heatmap of colonic index, CCR7^+^/CCR7^−^ T lymphocytes, MPO, and gut microflora. *n* = 5. *P ≤ 0.05, **P ≤ 0.01, ***P ≤ 0.001.

The aforementioned results suggested that COG was effective in reducing inflammation of the intestines and oxidative stress response and in regulating T lymphocyte subsets and gut microflora composition in colitic mice. However, whether COG regulates the correlation or consistency among the three factors has not been elucidated. Here, canonical correlation analysis (CCA), distance-based redundancy analysis (db-RDA), and Spearman’s correlation heatmap were used to analyze their correlation. In the CCA and db-RDA analyses, the points of different colors or shapes in the figure represent sample groups under different environments or conditions. The red arrows indicate quantitative environmental factors (such as colon weight index, MPO, etc.). The length of the arrows represents the impact of environmental factors on species data, and whether the direction of the arrow and the point is consistent represents positive and negative correlations. Based on the CCA analysis (**Figure 9A**), the abundance of colon weight index, MPO, and CD4^+^CD45^+^CCR7^+^ and CD4^+^CD45^+^CCR7^−^ cells was consistent with the gut microflora of the DSS group, which meant that these environmental factors were positively related with the gut microflora in the DSS group. db-RDA analysis is similar to PCoA analysis but is constrained by additional environmental factors. In the db-RDA graph at the OTU level (**Figure 9B**), the abundance of colon weight index and MPO was consistent with the gut microflora of the DSS group, which meant that these factors were positively related to the gut microflora in the DSS group at the OTU level. Pearson correlation analysis on species (**Figure 9C**) showed that *Akkermansia* was positively correlated with all the four factors, which indicated that *Akkermansia* may be involved in COG regulation of inflammation, oxidative stress, and T lymphocyte subsets. *Bacteroides* was positively correlated with CD4^+^CD45^+^CCR7^+^ and CD4^+^CD45^+^CCR7^−^ cells. In addition, *Alistipes* was negatively correlated with MPO and CD4^+^CD45^+^CCR7^+^ and CD4^+^CD45^+^CCR7^−^ cells, while *Lachnospiraceae_NK4A136_group* only had a negative correlation with colon weight index (**Figure 9C**).

## Discussion

*Chimonanthus nitens* Oliv. leaf granule is a common clinical medication for the treatment of respiratory diseases, which has been used as a recommended drug for the prevention and treatment of COVID-19 in Jiangxi, China (Wu et al., 2021). In this study, COG alleviated DSS-induced experimental colitis by reducing intestinal inflammatory injury and excessive oxidative stress and regulating the composition of Treg cells and gut microflora. By reviewing domestic and foreign literature, it was found that using COG to control UC, whether in humans or animals, was rarely reported. However, inspired by folk diagnosis and treatment experience, combined with the material basis and pharmacological activity of *C. nitens* Oliv. leaf, we proposed the hypothesis that COG can be used to treat DSS-induced colitis. The DSS-induced colitis model is a classic model for UC drug development. Our results showed that COG has a significant protective effect on DSS-induced colitis, as evidenced by the recovery of weight loss, DAI score, colon weight, colon length, and histopathological score. However, not all doses of COG showed significant effects. In terms of therapeutic effect, compared with the low-dose COG treatment, the high- and medium-dose COG treatments can better alleviate weight loss and increase DAI score in colitic mice. In the anti-inflammatory evaluation experiment, the regulatory effect of COG on inflammatory cytokines was generally dose-dependent, except that the low-dose COG did not effectively restore the level of IL-4 in colon tissue. Similarly, *in vitro*, 2 mg/ml of COG did not effectively inhibit the levels of IL-1β and TNF-α produced by LPS-induced RAW264.7 cells. It was possible that the low dose of COG or the duration of treatment used in this trial was not sufficient to affect DSS-induced UC. The results above suggested that the anti-inflammatory activity of COG is affected by drug dose, which provides a basis to further explore the dose–effect relationship of COG.

In IBD, intestinal long-term exposure to reactive oxygen species leads to intestinal oxidative stress injury, which is one of the common pathogenic factors of IBD (Ananthakrishnan et al., 2018). Studies have confirmed that compared with healthy people, the total antioxidant capacity of UC patients decreased significantly (Achitei et al., 2013), and adding antioxidants to the diet can effectively prevent the intestines from oxidative stress damage (Moura et al., 2015). In this study, COG significantly reduced the levels of NO, MDA, and MPO in colitic mice and improved the levels of MAO, SOD, GSH-Px, and eNOS, which contributed to UC remission. Antioxidant activity is one of the most important biological activities of *C. nitens* Oliv. leaf, which is closely related to a large number of antioxidant components, such as flavonoids (rutin, quercetin, kaempferol, etc.) and phenolic compounds (Wu et al., 2021). Sufficient experimental and clinical evidence has proven that *C. nitens* Oliv. leaf has strong antioxidant activity: First, the extracts (such as polysaccharides) from *C. nitens* Oliv. leaf can increase the enzyme activities of SOD, catalase (CAT), GSH-Px, and total antioxidant capacity (T-AOC) to eliminate oxidative stress injury (Chen et al., 2017a; Ye et al., 2020). Particularly, MAO activity was significantly reduced in the colon tissue of patients with active and quiescent colitis. Moreover, MAO activity was much lower in active colitis than in quiescent colitis (Borkje et al., 1987). Interestingly, COG upregulated MAO in our study, and we thought that it was possible to improve monoamine metabolism and the main source of ROS to protect the mucosa from injury. Second, COG can markedly decrease MPO to inhibit neutrophil infiltration (Rahal et al., 2014) or reduce MDA to suppress lipid peroxidation in experimental colitis induced by DSS. Finally, in the present study, COG can control the mucosa from oxidative stress injury induced by overexpressed NO products, which are increased when activated inflammatory cells stimulate inducible nitric oxide synthase (iNOS) to induce oxidative stress injury in experimental IBD (Pacher et al., 2007). Based on previous studies, the protective or damage-promoting effects of eNOS on mucosal inflammation had been disputed (Sasaki et al., 2003; Vallance et al., 2004). Combined with the above analysis, we found in our study that COG can effectively improve the eNOS level, which hinted that COG can beneficially protect the damaged colonic mucosa by inhibiting oxidative stress injury.

There is a material basis to support the anti-inflammatory and antioxidant effects of COG. The chemical constituents of *C. nitens* Oliv. leaf are complex, mainly including flavonoids, volatile oil, alkaloids, steroids, and coumarins. In this study, we found six main compounds of COG, namely, scopoletin, isofraxidin, scoparone, rutin, chimonanthine, and calycanthine, through LC-MS/MS material identification.

The main active ingredients of COG present definite pharmacological actions, including anti-inflammatory (such as scopoletin, scoparone, isofraxidin), antioxidant (such as scoparone, isofraxidin, rutin), regulatory of gut microflora (such as alkaloids and isofraxidin), and antitumor (such as isofraxidin, rutin) (Wang et al., 2017; Enogieru et al., 2018; Imani et al., 2020; Liu et al., 2020; Majnooni et al., 2020; Nouri et al., 2020; Sakthivel et al., 2021), which help alleviate colonic mucosa injury induced by DSS. Chimonanthine and calycanthine are important alkaloids in *C. nitens* Oliv. leaf. Notably, unlike the other components mentioned above, chimonanthine and calycanthine are special alkaloids of *Chimonanthus praecox*. Our results showed (**Table 2**) that the content of calycanthine in *C. nitens* Oliv. leaf granule is relatively high. In the present experiment, COG was proven to have the effect of relieving colitis in mice, which set the stage for the development of monomeric drugs from *C. nitens* Oliv. leaf for the treatment of UC in the future.

The mechanism of COG-treated colitis in mice involves immunoregulation of Treg or memory T cells and other T cells. Treg is a kind of immunosuppressive cells that participates in the pathogenesis of IBD in the intestinal microenvironment (Galvez, 2014), which controls intestinal inflammatory response mainly through the secretion of anti-inflammatory cytokines such as IL-10 and TGF-β (Mayne and Williams, 2013). The immunosuppressive effect of Treg cells on inflammatory sites requires chemokines such as CCR7 to promote their migration. CCR7 affects the function of Treg *in vivo* by regulating the transport of Treg cells to the lymph nodes and inflammatory sites. When Treg cells lack CCR7 expression, the migration into the lymph nodes is impaired and the suppressive effect is weakened. However, under inflammatory conditions, effectors and memory Treg, which are deficient in CCR7, accumulate at the inflammatory sites and show a stronger suppressive effect on inflammation (Menning et al., 2007). In this study, CD4^+^CCR7^+/−^ cells in mice with DSS-induced colitis were overactivated, and their subsets CD4^+^CCR7^+^IL-17A^+^ and CD4^+^CCR7^−^IL-17A^+^ increased significantly, while CD4^+^CCR7^+^IL-10^+^, CD4^+^CCR7^−^IL-10^+^, CD4^+^CCR7^+^Foxp3^+^, and CD4^+^CCR7^−^Foxp3^+^ decreased significantly. After COG treatment, the changes of CD4^+^CCR7^+/−^ cells and their subsets were effectively reversed. The balance between Th17 and Treg cells in CD4^+^CCR7^+/−^ cell subsets was restored, which effectively alleviated UC.

Local Treg is crucial to maintain gut microbiota balance. CCR7^-/-^ Treg cells alway accumulate in the effector or inflammatory local to exert immediate effect and exacerbate metabolic disorders of microflora. Gut flora imbalance is one of the important reasons involved in the pathogenesis of UC (Ni et al., 2017). Compared with healthy people, the α-diversity of gut flora in UC patients decreased significantly (Colquhoun et al., 2020). Intestinal bacteria, such as *Clostridium*, *Streptococcus*, *Bacteroides*, and *Escherichia coli*, produced proinflammatory mediators and indirectly activated the NF-κB signaling pathway under specific conditions, and the overactivated NF-κB signaling pathway reduced the apoptosis of inflammatory cells and immune cells (Vieira-Silva et al., 2019). In this study, the regulatory effect of COG on intestinal flora of UC mice was reported for the first time. COG effectively upregulated the relative abundance of *Lachnospiraceae_NK4A136_group*, *Alistipes*, and *Lachnospiraceae_UCG-006*, thus alleviating DSS-induced experimental colitis. Meanwhile, the correlation analysis showed that *Alistipes* was negatively correlated with MPO and CD4^+^CD45^+^CCR7^+^ and CD4^+^CD45^+^CCR7^+^ cells when experimental colitis was treated with COG. As a beneficial bacterium, *Alistipes* (such as *A. finegordii*) mainly existed in the gut of healthy people (Shkoporov et al., 2015) and effectively attenuated experimental colitis by gavage (Dziarski et al., 2016). Some studies indicated that patients with UC had lower amounts of *Lachnospiraceae* than healthy people, which was possible because the reduced abundance of *Lachnospiraceae* produced low butyrogenesis and thus triggered the recurrence of UC (Frank et al., 2007; Vacca et al., 2020). We deduced that COG can improve the balance of CD4^+^CD45^+^CCR7^+^/CD4^+^CD45^+^CCR7^−^ cells and CCR7^+^/CCR7^−^ Treg cells to maintain immunity homeostasis in the intestinal tract, further restoring the above beneficial bacterium level to rebuild the balance of gut microbiota in colitis. Of course, this needs further research to verify the relationship between COG, CCR7^+^/CCR7^−^ Treg cells, and gut microbiota.

Some studies have shown that the extract from *C. nitens* Oliv. leaf regulates glucose transporters and reduces blood sugar by affecting the glycolipid metabolism in diabetic mice (Chen et al., 2018), which hints that COG can regulate cell metabolism (such as glucose metabolism and fat metabolism). In UC treatment and research, it is essential to regulate the function and differentiation of Treg cells by metabolism improvement (Cluxton et al., 2019; Galgani et al., 2021). Hence, we hypothesize that COG can improve the immune metabolism of CCR7^+^/CCR7^−^ Treg cells to restore the balance of gut microbiota and finally alleviate UC. In the future, CCR7^+^/CCR7^−^ Treg cells from colitic mice will be separated and cultured with COG and then their energy metabolism and differentiation level will be analyzed under special microbiota (such as *Alistipes* and *Lachnospiraceae*). Furthermore, colitic mice without special microbiota (such as *Alistipes* and *Lachnospiraceae*) will be prepared and treated with COG, and we will measure the function and metabolism of CCR7^+^/CCR7^−^ Treg cells separated by flow cytometry, Seahorse cell energy analysis system, and so on. All these endeavors will lead to a better understanding of the mechanism of COG to regulate the relationship between CCR7^+^/CCR7^−^ Treg cells and gut microbiota in UC treatment.

In conclusion, we found for the first time that *C. nitens* Oliv. leaf granule alleviates DSS-induced experimental colitis through remitting inflammation *in vivo* and *in vitro*, reducing oxidative stress response, regulating Treg cell differentiation, and improving intestinal flora composition. These findings should help us to explore the potential of COG as an alternative treatment for UC and expand the application prospect of COG in the clinic.

## Data Availability Statement

The original contributions presented in the study are included in the article/supplementary material. Further inquiries can be directed to the corresponding authors.

## Ethics Statement

The animal study was reviewed and approved by The Animal Care and Use Committee of Jiangxi University of Traditional Chinese Medicine.

## Author Contributions

J-QH: conceptualization, investigation, writing—review and editing, and funding acquisition. S-YW: methodology, formal analysis, and data curation. NC, Y-BZ, F-HY, and M-DL: investigation. D-YL: provision of resources, supervision, project administration, and funding acquisition. H-MZ and S-SL: conceptualization, visualization, project administration, and funding acquisition. All authors contributed to the article and approved the submitted version.

## Funding

This study was supported by the National Natural Science Foundation of China (Grant No. 81860792) and the National College Students Innovation and Entrepreneurship Training Program of China (Grant Nos. 202013437001, 202110412003, 202113437002).

## Conflict of Interest

The authors declare that the research was conducted in the absence of any commercial or financial relationships that could be construed as a potential conflict of interest.

## Publisher’s Note

All claims expressed in this article are solely those of the authors and do not necessarily represent those of their affiliated organizations, or those of the publisher, the editors and the reviewers. Any product that may be evaluated in this article, or claim that may be made by its manufacturer, is not guaranteed or endorsed by the publisher.

## References

[B1] Achitei D. Ciobica A. Balan G. Gologan E. Stanciu C. Stefanescu G. (2013). Different Profile of Peripheral Antioxidant Enzymes and Lipid Peroxidation in Active and non-Active Inflammatory Bowel Disease Patients. Dig Dis. Sci. 58, 1244–1249. doi: 10.1007/s10620-012-2510-z 23306840

[B2] Adjouzem C. F. Gilbert A. Mbiantcha M. Yousseu Nana W. Matah Marthe Mba V. Djuichou Nguemnang S. F.. (2020). Effects of Aqueous and Methanolic Extracts of Stem Bark of Alstonia Boonei De Wild. (Apocynaceae) on Dextran Sodium Sulfate-Induced Ulcerative Colitis in Wistar Rats. Evid Based Complement Alternat Med. 2020, 4918453. doi: 10.1155/2020/4918453 32565862 PMC7277065

[B3] Ananthakrishnan A. N. Bernstein C. N. Iliopoulos D. Macpherson A. Neurath M. F. Ali R. A. R.. (2018). Environmental Triggers in IBD: A Review of Progress and Evidence. Nat. Rev. Gastroenterol. Hepatol. 15, 39–49. doi: 10.1038/nrgastro.2017.136 29018271

[B4] Borkje B. Laerum O. D. Schrumpf E. (1987). Enzyme Activities in Biopsy Specimens From Large-Bowel Mucosa in Ulcerative Colitis. Scand. J. Gastroenterol. 22, 443–448. doi: 10.3109/00365528708991488 3602924

[B5] Chen H. Jiang Y. Yang Z. Hu W. Xiong L. Wang N.. (2017a). Effects of Chimonanthus Nitens Oliv. Leaf Extract on Glycolipid Metabolism and Antioxidant Capacity in Diabetic Model Mice. Oxid. Med. Cell Longev 2017, 7648505. doi: 10.1155/2017/7648505 29057036 PMC5625751

[B6] Chen H. Ouyang K. Jiang Y. Yang Z. Hu W. Xiong L.. (2017b). Constituent Analysis of the Ethanol Extracts of Chimonanthus Nitens Oliv. Leaves and Their Inhibitory Effect on Alpha-Glucosidase Activity. Int. J. Biol. Macromol 98, 829–836. doi: 10.1016/j.ijbiomac.2017.02.044 28223131

[B7] Chen H. Xiong L. Wang N. Liu X. Hu W. Yang Z.. (2018). Chimonanthus Nitens Oliv. Leaf Extract Exerting Anti-Hyperglycemic Activity by Modulating GLUT4 and GLUT1 in the Skeletal Muscle of a Diabetic Mouse Model. Food Funct. 9, 4959–4967. doi: 10.1039/C8FO00954F 30182103

[B8] Cluxton D. Petrasca A. Moran B. Fletcher J. M. (2019). Differential Regulation of Human Treg and Th17 Cells by Fatty Acid Synthesis and Glycolysis. Front. Immunol. 10, 115. doi: 10.3389/fimmu.2019.00115 30778354 PMC6369198

[B9] Colquhoun C. Duncan M. Grant G. (2020). Inflammatory Bowel Diseases: Host-Microbial-Environmental Interactions in Dysbiosis. Diseases 8, 13. doi: 10.3390/diseases8020013 PMC7348996 32397606

[B10] Dziarski R. Park S. Y. Kashyap D. R. Dowd S. E. Gupta D. (2016). Pglyrp-Regulated Gut Microflora Prevotella Falsenii, Parabacteroides Distasonis and Bacteroides Eggerthii Enhance and Alistipes Finegoldii Attenuates Colitis in Mice. PloS One 11, e0146162. doi: 10.1371/journal.pone.0146162 26727498 PMC4699708

[B11] Enogieru A. B. Haylett W. Hiss D. C. Bardien S. Ekpo O. E. (2018). Rutin as a Potent Antioxidant: Implications for Neurodegenerative Disorders. Oxid. Med. Cell. Longevity. 2018, 624107. doi: 10.1155/2018/6241017 PMC6040293 30050657

[B12] Frank D. N. St Amand A. L. Feldman R. A. Boedeker E. C. Harpaz N. Pace N. R. (2007). Molecular-Phylogenetic Characterization of Microbial Community Imbalances in Human Inflammatory Bowel Diseases. Proc. Natl. Acad. Sci. U.S.A. 104, 13780–13785. doi: 10.1073/pnas.0706625104 17699621 PMC1959459

[B13] Galgani M. Bruzzaniti S. La Rocca C. Micillo T. de Candia P. Bifulco M.. (2021). Immunometabolism of Regulatory T Cells in Cancer. Mol. Aspects Med. 77, 100936. doi: 10.1016/j.mam.2020.100936 33250195

[B14] Galvez J. (2014). Role of Th17 Cells in the Pathogenesis of Human IBD. ISRN Inflammation 2014, 928461. doi: 10.1155/2014/928461 25101191 PMC4005031

[B15] Grayson M. H. Camarda L. E. Hussain S.-R. A. Zemple S. J. Hayward M. Lam V.. (2018). Intestinal Microbiota Disruption Reduces Regulatory T Cells and Increases Respiratory Viral Infection Mortality Through Increased Ifnγ Production. Front. Immunol. 9. doi: 10.3389/fimmu.2018.01587 PMC6048222 30042764

[B16] Hansen J. Gulati A. Sartor R. B. (2010). The Role of Mucosal Immunity and Host Genetics in Defining Intestinal Commensal Bacteria. Curr. Opin. Gastroenterol. 26, 564–571. doi: 10.1097/MOG.0b013e32833f1195 20871399 PMC3733357

[B17] Iheozor-Ejiofor Z. Kaur L. Gordon M. Baines P. A. Sinopoulou V. Akobeng A. K. (2020). Probiotics for Maintenance of Remission in Ulcerative Colitis. Cochrane Database Systematic Rev. 2020 (3), CD007443. doi: 10.1002/14651858.CD007443.pub3. Accessed 06 June 2022. PMC7059960 32128794

[B18] Imani A. Maleki N. Bohlouli S. Kouhsoltani M. Sharifi S. Maleki Dizaj S. (2021). Molecular Mechanisms of Anticancer Effect of Rutin. Phytotherapy Research. 35(5), 2500–2513. doi: 10.1002/ptr.6977 33295678

[B19] Ji J. Ge X. Chen Y. Zhu B. Wu Q. Zhang J.. (2019). Daphnetin Ameliorates Experimental Colitis by Modulating Microbiota Composition and Treg/Th17 Balance. FASEB J. 33, 9308–9322. doi: 10.1096/fj.201802659RR 31145641

[B20] Kamikozuru K. Fukunaga K. Hirota S. Hida N. Ohda Y. Yoshida K.. (2009). The Expression Profile of Functional Regulatory T Cells, CD4+ CD25high+/forkhead Box Protein P3+, in Patients With Ulcerative Colitis During Active and Quiescent Disease. Clin. Exp. Immunol. 156, 320–327. doi: 10.1111/j.1365-2249.2009.03904.x 19292766 PMC2759481

[B21] Kaplan G. G. Windsor J. W. (2021). The Four Epidemiological Stages in the Global Evolution of Inflammatory Bowel Disease. Nat. Rev. Gastroenterol. Hepatol. 18, 56–66. doi: 10.1038/s41575-020-00360-x 33033392 PMC7542092

[B22] Korhonen R. Lahti A. Kankaanranta H. Moilanen E. (2005). Nitric Oxide Production and Signaling in Inflammation. Curr. Drug Targets Inflammation Allergy 4, 471–479. doi: 10.2174/1568010054526359 16101524

[B23] Li F. Han Y. Cai X. Gu M. Sun J. Qi C.. (2020). Dietary Resveratrol Attenuated Colitis and Modulated Gut Microbiota in Dextran Sulfate Sodium-Treated Mice. Food Funct. 11, 1063–1073. doi: 10.1039/C9FO01519A 31825043 PMC7122795

[B24] Li X. Song P. Li J. Tao Y. Li G. Li X.. (2017). The Disease Burden and Clinical Characteristics of Inflammatory Bowel Disease in the Chinese Population: A Systematic Review and Meta-Analysis. Int. J. Environ. Res. Public Health 14 (3), 238. doi: 10.3390/ijerph14030238 PMC5369074 28264519

[B25] Liu B. Deng X. Jiang Q. Li G. Zhang J. Zhang N.. (2020). Scoparone Improves Hepatic Inflammation and Autophagy in Mice With Nonalcoholic Steatohepatitis by Regulating the ROS/P38/Nrf2 Axis and PI3K/AKT/mTOR Pathway in Macrophages. BioMed. Pharmacother. 125, 109895. doi: 10.1016/j.biopha.2020.109895 32000066

[B26] Liu Y. Liu X. Hua W. Wei Q. Fang X. Zhao Z.. (2018). Berberine Inhibits Macrophage M1 Polarization *via* AKT1/SOCS1/NF-kappaB Signaling Pathway to Protect Against DSS-Induced Colitis. Int. Immunopharmacol 57, 121–131. doi: 10.1016/j.intimp.2018.01.049 29482156

[B27] Majnooni M. B. Fakhri S. Shokoohinia Y. Mojarrab M. Kazemi-Afrakoti S. Farzaei M. H. (2020). Isofraxidin: Synthesis, Biosynthesis, Isolation, Pharmacokinetic and Pharmacological Properties. Molecules (Basel, Switzerland) 25 (9), 2040. doi: 10.3390/molecules25092040 PMC7248759 32349420

[B28] Mayne C. G. Williams C. B. (2013). Induced and Natural Regulatory T Cells in the Development of Inflammatory Bowel Disease. Inflammation Bowel Dis. 19, 1772–1788. doi: 10.1097/MIB.0b013e318281f5a3 PMC3690174 23656897

[B29] Menning A. Hopken U. E. Siegmund K. Lipp M. Hamann A. Huehn J. (2007). Distinctive Role of CCR7 in Migration and Functional Activity of Naive- and Effector/Memory-Like Treg Subsets. Eur. J. Immunol. 37, 1575–1583. doi: 10.1002/eji.200737201 17474155

[B30] Moura F. A. de Andrade K. Q. Dos Santos J. C. F. Araujo O. R. P. Goulart M. O. F. (2015). Antioxidant Therapy for Treatment of Inflammatory Bowel Disease: Does it Work? Redox Biol. 6, 617–639. doi: 10.1016/j.redox.2015.10.006 26520808 PMC4637335

[B31] Nakase H. Uchino M. Shinzaki S. Matsuura M. Matsuoka K. Kobayashi T.. (2021). Evidence-Based Clinical Practice Guidelines for Inflammatory Bowel Disease 2020. J. Gastroenterol. 56, 489–526. doi: 10.1007/s00535-021-01784-1 33885977 PMC8137635

[B32] Ni J. Wu G. D. Albenberg L. Tomov V. T. (2017). Gut Microbiota and IBD: Causation or Correlation? Nat. Rev. Gastroenterol. Hepatol. 14, 573–584. doi: 10.1038/nrgastro.2017.88 28743984 PMC5880536

[B33] Nouri Z. Fakhri S. Nouri K. Wallace C. E. Farzaei M. H. Bishayee A. (2020). Targeting Multiple Signaling Pathways in Cancer: The Rutin Therapeutic Approach. Cancers (Basel) 12 (8), 2276. doi: 10.3390/cancers12082276 PMC7463935 32823876

[B34] Oladele J. O. Ajayi E. I. Oyeleke O. M. Oladele O. T. Olowookere B. D. Adeniyi B. M. (2020). Curative Potential of Nigerian Medicinal Plants in COVID-19 Treatment: A Mechanistic Approach. Jordan J. Biol. Sci. 13.

[B35] Oldstone M. B. Rosen H. (2014). Cytokine Storm Plays a Direct Role in the Morbidity and Mortality From Influenza Virus Infection and is Chemically Treatable With a Single Sphingosine-1-Phosphate Agonist Molecule. Curr. Top. Microbiol. Immunol. 378, 129–147. doi: 10.1007/978-3-319-05879-5_6 24728596 PMC7121493

[B36] Pacher P. Beckman J. S. Liaudet L. (2007). Nitric Oxide and Peroxynitrite in Health and Disease. Physiol. Rev. 87, 315–424. doi: 10.1152/physrev.00029.2006 17237348 PMC2248324

[B37] Pavlick K. P. Laroux F. S. Fuseler J. Wolf R. E. Gray L. Hoffman J.. (2002). Role of Reactive Metabolites of Oxygen and Nitrogen in Inflammatory Bowel Disease. Free Radic. Biol. Med. 33, 311–322. doi: 10.1016/S0891-5849(02)00853-5 12126753

[B38] Peng J. Zheng T. T. Li X. Liang Y. Wang L. J. Huang Y. C.. (2019). Plant-Derived Alkaloids: The Promising Disease-Modifying Agents for Inflammatory Bowel Disease. Front. Pharmacol. 10, 351. doi: 10.3389/fphar.2019.00351 31031622 PMC6473079

[B39] Rahal A. Kumar A. Singh V. Yadav B. Tiwari R. Chakraborty S.. (2014). Oxidative Stress, Prooxidants, and Antioxidants: The Interplay. BioMed. Res. Int. 2014, 761264. doi: 10.1155/2014/761264 24587990 PMC3920909

[B40] Reinhardt R. L. Khoruts A. Merica R. Zell T. Jenkins M. K. (2001). Visualizing the Generation of Memory CD4 T Cells in the Whole Body. Nature 410, 101–105. doi: 10.1038/35065111 11242050

[B41] Sakthivel K. M. Vishnupriya S. Priya Dharshini L. C. Rasmi R. R. Ramesh B. (2021). Modulation of Multiple Cellular Signalling Pathways as Targets for Anti-Inflammatory and Anti-Tumorigenesis Action of Scopoletin. J. Pharm. Pharmacol. 74, 147–161. doi: 10.1093/jpp/rgab047 33847360

[B42] Sallusto F. Lenig D. Forster R. Lipp M. Lanzavecchia A. (1999). Two Subsets of Memory T Lymphocytes With Distinct Homing Potentials and Effector Functions. Nature 401, 708–712. doi: 10.1038/44385 10537110

[B43] Sartor R. B. (2006). Mechanisms of Disease: Pathogenesis of Crohn's Disease and Ulcerative Colitis. Nat. Clin. Pract. Gastroenterol. Hepatol. 3, 390–407. doi: 10.1038/ncpgasthep0528 16819502

[B44] Sasaki M. Bharwani S. Jordan P. Elrod J. W. Grisham M. B. Jackson T. H.. (2003). Increased Disease Activity in eNOS-Deficient Mice in Experimental Colitis. Free Radic. Biol. Med. 35, 1679–1687. doi: 10.1016/j.freeradbiomed.2003.09.016 14680690

[B45] Shkoporov A. N. Chaplin A. V. Khokhlova E. V. Shcherbakova V. A. Motuzova O. V. Bozhenko V. K.. (2015). Alistipes Inops Sp. Nov. And Coprobacter Secundus Sp. Nov., Isolated From Human Faeces. Int. J. Syst. Evol. Microbiol. 65, 4580–4588. doi: 10.1099/ijsem.0.000617 26377180

[B46] Shu R. G. Wan Y. L. Wang X. M. (2019). Non-Volatile Constituents and Pharmacology of Chimonanthus: A Review. Chin. J. Nat. Med. 17, 161–186. doi: 10.1016/S1875-5364(19)30020-2 30910054

[B47] Takahashi M. Nakamura K. Honda K. Kitamura Y. Mizutani T. Araki Y.. (2006). An Inverse Correlation of Human Peripheral Blood Regulatory T Cell Frequency With the Disease Activity of Ulcerative Colitis. Digestive Dis. Sci. 51, 677–686. doi: 10.1007/s10620-006-3191-2 16614988

[B48] Vacca M. Celano G. Calabrese F. M. Portincasa P. Gobbetti M. De Angelis M. (2020). The Controversial Role of Human Gut Lachnospiraceae. Microorganisms 8 (4), 573. doi: 10.3390/microorganisms8040573 PMC7232163 32326636

[B49] Vallance B. A. Dijkstra G. Qiu B. van der Waaij L. A. van Goor H. Jansen P. L.. (2004). Relative Contributions of NOS Isoforms During Experimental Colitis: Endothelial-Derived NOS Maintains Mucosal Integrity. Am. J. Physiol. Gastrointest Liver Physiol. 287, G865–G874. doi: 10.1152/ajpgi.00187.2004 15217783

[B50] Vezza T. Rodriguez-Nogales A. Algieri F. Utrilla M. P. Rodriguez-Cabezas M. E. Galvez J. (2016). Flavonoids in Inflammatory Bowel Disease: A Review. Nutrients 8, 211. doi: 10.3390/nu8040211 27070642 PMC4848680

[B51] Vich Vila A. Imhann F. Collij V. Jankipersadsing S. A. Gurry T. Mujagic Z.. (2018). Gut Microbiota Composition and Functional Changes in Inflammatory Bowel Disease and Irritable Bowel Syndrome. Sci. Transl. Med. 10, eaap8914. doi: 10.1126/scitranslmed.aap8914 30567928

[B52] Vieira-Silva S. Sabino J. Valles-Colomer M. Falony G. Kathagen G. Caenepeel C.. (2019). Quantitative Microbiome Profiling Disentangles Inflammation- and Bile Duct Obstruction-Associated Microbiota Alterations Across PSC/IBD Diagnoses. Nat. Microbiol. 4, 1826–1831. doi: 10.1038/s41564-019-0483-9 31209308

[B53] Wang Y. Wang M. Chen B. Shi J. (2017). Scoparone Attenuates High Glucose-Induced Extracellular Matrix Accumulation in Rat Mesangial Cells. Eur. J. Pharmacol. 815, 376–380. doi: 10.1016/j.ejphar.2017.09.039 28970015

[B54] Wirtz S. Popp V. Kindermann M. Gerlach K. Weigmann B. Fichtner-Feigl S.. (2017). Chemically Induced Mouse Models of Acute and Chronic Intestinal Inflammation. Nat. Protoc. 12, 1295–1309. doi: 10.1038/nprot.2017.044 28569761

[B55] Wu C.-C. Chen J.-S. Wu W.-M. Liao T.-N. Chu P. Lin S.-H.. (2005). Myeloperoxidase Serves as a Marker of Oxidative Stress During Single Haemodialysis Session Using Two Different Biocompatible Dialysis Membranes. Nephrol. Dialysis Transplant. 20, 1134–1139. doi: 10.1093/ndt/gfh764 15814542

[B56] Wu Y. Liu Y. Lin L. Luo Y. Shi G. Li H. (2021). Research Progress on Chemical Composition, Preparation, Pharmacological Action and Clinical Application of Chimonanthus Nitens Folium. China J. Traditional Chin. Med. Pharm. 36, 6599–6607. doi: CNKI:SUN:BXYY.0.2021-11-079

[B57] Xavier R. J. Podolsky D. K. (2007). Unravelling the Pathogenesis of Inflammatory Bowel Disease. Nature 448, 427–434. doi: 10.1038/nature06005 17653185

[B58] Ye X. An Q. Chen S. Liu X. Wang N. Li X.. (2020). The Structural Characteristics, Antioxidant and Hepatoprotection Activities of Polysaccharides From Chimonanthus Nitens Oliv. Leaves Int. J. Biol. Macromol 156, 1520–1529. doi: 10.1016/j.ijbiomac.2019.11.200 31783077

[B59] Zhang X. Xu M. Zhang J. Wu L. Liu J. Si J. (2017). Identification and Evaluation of Antioxidant Components in the Flowers of Five Chimonanthus Species. Ind. Crops Products 102, 164–172. doi: 10.1016/j.indcrop.2017.03.014

